# Accelerometer assessed moderate-to-vigorous physical activity and successful ageing: results from the Whitehall II study

**DOI:** 10.1038/srep45772

**Published:** 2017-04-03

**Authors:** Mehdi Menai, Vincent T. van Hees, Alexis Elbaz, Mika Kivimaki, Archana Singh-Manoux, Séverine Sabia

**Affiliations:** 1 Centre for Research in Epidemiology and Population Health, Univ. Paris-Sud, UVSQ, INSERM U1018, Université Paris-Saclay, Villejuif, France; 2 Netherlands eScience Center, Amsterdam, The Netherland; 3 Department of Epidemiology and Public Health, University College London, London, United Kingdom

## Abstract

Physical activity is key for successful ageing, but questions remain regarding the optimal physical activity pattern. We examined the cross-sectional association between physical activity and successful ageing using data on 3,749 participants (age range = 60–83years) of the Whitehall II study. The participants underwent a clinical assessment, completed a 20-item physical activity questionnaire, and wore a wrist-mounted accelerometer for 9 days. Successful ageing was defined as good cognitive, motor, and respiratory functioning, along with absence of disability, mental health problems, and major chronic diseases. Time spent in moderate-to-vigorous physical activity (MVPA) episodes assessed by accelerometer was classified as “short” (1–9.59 minutes) and “long” (≥10 minutes) bouts. Linear multivariate regression showed that successful agers (N = 789) reported 3.79 (95% confidence interval (CI): 1.39–6.19) minutes more daily MVPA than other participants. Accelerometer data showed this difference to be 3.40 (95% CI:2.44–4.35) minutes for MVPA undertaken in short bouts, 4.16 (95% CI:3.11–5.20) minutes for long bouts, and 7.55 (95% CI:5.86–9.24) minutes for all MVPA bouts lasting 1 minute or more. Multivariate logistic regressions showed that participants undertaking ≥150 minutes of MVPA per week were more likely to be successful agers with both self-reported (Odd Ratio (OR) = 1.29,95% (CI):1.09–1.53) and accelerometer data (length bout ≥1 minute:OR = 1.92, 95%CI:1.60–2.30). Successful agers practice more MVPA, having both more short and long bouts, than non-successful agers.

More than one in ten adults globally is aged 60 years or older and this proportion is predicted to almost double by 2050^1^. Population ageing raises economic and societal challenges that stem from increased burden of multimorbidity and disability^1^. It also emphasizes the need for better understanding of preventive or curative strategies that would allow individuals to live free of disease longer and in good functional health^2^. Multiple risk and protective factors are associated with successful ageing^3^,^4^. One of them is physical activity^4^ which reduces risk of diabetes, cardiovascular diseases and cancers, and may also be neuroprotective^2^,^5^. The World Health Organization recommends that older adults practice at least 150 minutes of moderate-to-vigorous physical activity (MVPA) per week, made up of bouts lasting a minimum 10 minutes^6^.

There is considerable evidence linking physical activity to successful ageing in cross-sectional^7^,^8^,^9^ and longitudinal studies^10^,^11^,^12^,^13^. However, this evidence is based on self-reported physical activity which is prone to reporting biases. Recent studies using objective measures of physical activity, such as accelerometers, have mostly examined associations of health with total duration of physical activity. These studies found more physical activity^14^,^15^,^16^ or MVPA^15^,^17^ to be associated with favorable cardiometabolic outcomes^18^,^19^ and better motor^15^,^16^ and cognitive^14^,^17^ function. There is also some evidence to suggest that time spent in both short and long MVPA bouts is associated with better cardio-metabolic health^20^,^21^,^22^ and reduced risk of multimorbidity^23^. However, the importance of physical activity patterns, such as the duration of physical activity bouts and their intensity, for successful ageing has not yet been examined.

Our objective is to compare physical activity, assessed via questionnaire and accelerometer, between successful and non-successful agers using data from a well characterized cohort of community dwelling older adults, aged 60 to 83 years. We also compared the proportion of successful agers in physical activity groups defined by duration in MVPA bouts, and length and intensity of MVPA bouts.

## Methods

### Study population

We used data from the Whitehall II cohort study, established in 1985/1988 on 10,308 British civil servants (67% men) aged 35–55 years^24^. Informed consent was obtained from all participants. The study was approved by the ethics committee of University College London (reference number 85/0938) and conducted in accordance with the 2008 Helsinki Declaration. The study design consists of a clinical examination every 3 to 6 years since study inception. Accelerometer measurement was added to the study at the 2012/2013 wave of data collection for participants seen at the central London clinic and for those living in the South-Eastern regions of England who had their clinical assessment at home. For logistical reasons (charging and configuration of the devices), participants seen at home who resided in other parts of UK could not be offered the accelerometer measurement.

Whitehall II data, protocols, and other metadata are available to the scientific community. Please refer to the Whitehall II data sharing policy at http://www.ucl.ac.uk/whitehallII/data-sharing.

### Physical Activity

Physical activity was assessed in 2012/2013 using questionnaire and accelerometer.

#### Questionnaire

Physical activity was assessed using a modified version of the validated Minnesota leisure-time physical activity questionnaire^25^,^26^, including 20-items on frequency and duration of participation in different physical activities (e.g., walking, cycling, sports). Participants were requested to take into account activity patterns over the past four weeks, at work and leisure. For each activity, including the open-ended items, we assigned a metabolic equivalent (MET) value by using the latest version of the compendium of activity energy costs^27^. One MET value reflects the intensity of the activity relative to lying quietly. All activities with MET values of 3 or above were coded as MVPA and summed to yield total numbers per week.

#### Accelerometer

At the clinical examination (on average 5 days after completion of the questionnaire), participants with no contraindications (allergies to plastic or metal, traveling abroad the following week) were asked to wear a triaxial accelerometer (GENEActiv Original; Activinsights Ltd, Kimbolton, Cambs, UK, http://www.geneactiv.org/) on their non-dominant wrist for 9 consecutive, 24-hour, days. Acceleration was recorded at 85.7 Hz (corresponding to around 86 measurements per second) and as in previous studies using devices recording raw accelerometry^28^,^29^,^30^,^31^, acceleration was expressed relative to gravity (*g* units; 1 *g* = 9.81 m.s^−2^; 1 m*g* = 0.00981 m.s^−2^)). Calibration error was estimated based on static periods in the data and corrected if necessary (calibration correction range = 0.8 to 10.0 m*g,* mean correction = 2.5 m*g*)^32^. Next, the Euclidean Norm Minus One (ENMO), was used to quantify the acceleration related to the movement registered and expressed in milligravity (m*g*)^33^. Negative values were rounded to zero and ENMO values averaged over 5 second epochs.

As part of the accelerometer measurement, sleep periods were detected using a previously validated algorithm^34^. Data from the first waking to waking on the penultimate day were extracted, corresponding to 7 full days. For the present analysis, only data over the day (periods between waking and sleep onset) were used. Participants were included in the analysis if they had valid data, defined as daily wear time ≥2/3 of waking hours, for at least 2 weekdays and 2 week-end days. Non-wear time was estimated using a previously reported algorithm^33^ and was replaced by personalized mean value for each participant, calculated from their data on other days at the same time of day^31^,^35^.

In order for physical activity to be classified as MVPA, mean acceleration in the 5s-epoch (ENMO) needed to be at or above 100 m*g*^28^,^29^,^30^,^36^,^37^. To remove signals related to random wrist movement, we only retained activities lasting at least 1 minute for which 80% of the activity satisfied the 100 m*g* threshold criteria. In order to test the importance of length of a bout of physical activity, we identified bouts lasting 1 to 9 minutes 59 seconds and those lasting 10 minutes or more, hereafter labeled “short” and “long” bouts^20^,^21^ (further details in Supplementary Methods). Time spent in MVPA bouts lasting 1 minute or more was calculated as the sum of short and long bouts. The daily accelerometer-assessed time in MVPA over a week was calculated as the mean of measures over 7 days. For participants with less than 7 valid days of data, the following formula was used to standardize measurement to one week for all participants: [(5 × mean daily weekday MVPA time + 2 × mean daily week-end MVPA time)]/7. Accelerometer data were processed in R using the GGIR package (cran.r-project.org/web/packages/GGIR/index.html).

### Assessment of successful ageing

We used data from the study baseline (1985/1988) to 2012/2013 to ascertain successful ageing. As in previous studies, we used a comprehensive definition of successful ageing that included better function (free of disability, good mental health and better cognitive, motor and respiratory function) and absence of major chronic disease (coronary artery disease, stroke, diabetes, cancer and neurodegenerative disease)^13^,^38^. Chronic diseases were assessed from 1985/1988 to 2012/2013. Coronary heart disease, including myocardial infarction and definite angina, and stroke events were identified using linkage to national hospital records. Diabetes mellitus was defined as fasting blood glucose level of 7.0 mmol/L or more, HbA1c ≥6.5% (48 mmol/mol) based on blood sample assessed at the clinical examination, self-reported doctor-diagnosed diabetes, or the use of medications for diabetes. Cases of cancer were determined using the National Health Service’s cancer register. Neurodegenerative diseases included Parkinson’s disease and dementia and were assessed using linkage to national hospital records and mental health service dataset.

Functioning outcomes were assessed at the 2012/2013 wave of data collection. Disability was assessed based on participants’ responses to 6 questions on perceived difficulties in basic activities of daily living lasting for at least 3 months (dressing, walking, bathing/showering, eating, getting in or out of bed, using the toilet), with difficulties in one or more activities taken to signal disability. Mental health was assessed using the Center for Epidemiologic Studies Depression Scale questionnaire, a 20-items questionnaire designed to assess prevalence of depressive symptoms^39^, with scores below the threshold of 16 seen to indicate good mental health^39^. We defined better cognitive, motor and respiratory functioning as scores above the sex- and age-standardized tertile (i.e. above the 33.3^th^ percentile) for each measure. Cognitive functioning was assessed using a score of global cognition calculated as a standardized score of performances in 4 cognitive domains (short-term memory, attention (Trail-Making Test A), executive function (composite score of the Alice-Heim 4 and the Trail-Making Test B) and fluency (semantic and phonemic fluency tests)); motor function using walking speed measured over an 8-footlong course and respiratory function using forced expiratory volume in 1 second (FEV_1_) divided by height squared.

### Covariates

Sociodemographic variables were assessed by questionnaire in 2012/2013 and included age, sex, ethnicity (white and non-white), occupational position at age 50 years (high, intermediate or low, representing income and status at work) and education (university degree or lower). Health behaviours were also assessed by questionnaire. Smoking status was defined as current, past, and never smokers. Alcohol consumption was assessed using a question on the quantity of alcohol consumed in the previous week and was classified as no alcohol consumption in the previous week, moderate alcohol consumption (1–14 units/week in women and 1–21 units/week in men), and heavy drinkers (14 + units in women and 21 + units in men). The frequency of fruit and vegetable consumption was assessed on a 9- point scale, ranging from “seldom or never” to “4 or more times a day”, and was categorized as less than daily, once per day, or more than once per day. Season of data collection via accelerometer for each participant (winter: 21^st^ of December to 20^th^ of March, spring: 21^st^ of March to 20^th^ of June, summer: 21^st^ of June to 20^th^ of September, and autumn: 21^st^ of September to 20^th^ of December) was also included as a covariate.

### Statistical analyses

Two sets of analyses were undertaken. One, multivariate linear regression was used to compare mean MVPA duration (dependent variable) between successful agers and all others (independent variables). These analyses were repeated to study differences in mean acceleration, a measure of intensity, during MVPA bouts. Two, in order to compare the proportion of successful agers in different groups of physical activity patterns, the association of different physical activity characteristics (independent variable) with successful ageing (dependent variable) was examined using multivariate logistic regression and results expressed as odds ratios (OR) with 95% confidence intervals (CI). We used cubic splines to explore the shape of the association between MVPA and odds of successful ageing. All analyses, apart from the spline regressions, were adjusted for age, sex, ethnicity, education, smoking status, alcohol consumption, fruit and vegetable consumption, season and accelerometer wear time, with variables entered as categories as described in the Covariates section, apart from age which was entered as a continuous variable.

Sensitivity analyses were conducted to assess whether: 1) associations with MVPA were similar for components used to construct the successful ageing phenotype; 2) findings were similar when a more stringent cut-off of 120 m*g* was used to define MVPA using accelerometer data; 3) study findings could be explained partly by reverse causation by excluding from the analysis participants with disability and those who died within two years of the 2012/2013 data collection.

For all analyses, the statistical significance level was set at 0.05 and all tests were two-sided. We used Stata 13 (StataCorp LP, College Station, TX) for cubic splines regression and graphics; all others statistical analyses were performed using SAS software (version 9.4, SAS Institute Inc., Cary, NC, USA).

## Results

### Population selection

Of the 6,308 participants seen at the 2012–2013 wave of data collection, accelerometers were proposed to 4,880 participants (4,680 seen at the London clinic and 200 at home). Among them, 210 had contraindications, 4,282 (87.7%) consented, and 3,953 participants had valid accelerometer data. Data on physical activity questionnaire were available for 3,929 of these participants. In addition, chronic disease and functioning measurements for the successful ageing outcome were available on 3,773 of these participants. In total, 3,749 participants had information on successful ageing status, accelerometer and questionnaire physical activity data and covariates (flow chart in Supplementary Figure S1).

Compared to those not included in the analysis (n = 2,559), the analytic sample included participants who were younger (69.4 vs. 70.6 years, P < 0.001), more likely to be men (74.1% vs. 65.0%, P < 0.001) or from the lowest occupational position (10.8% vs. 15.8%, P < 0.001). The participants included in the analysis were also more likely to report ≥150 minutes of MVPA per week (52.7% vs. 47.6%, P < 0.001) and to qualify as successful agers (21.1% vs. 13.0%, P < 0.001).

### Study population characteristics

Among participants included in the analysis, 96.1% (N = 3,603) had valid accelerometer data for 7 days, 2.6% (N = 98) for 6 days, and 1.3% (N = 48) for 4–5 days. Missing data were replaced for 1–2 hours over the full observational period for 24.6% of the participants, 2–5 hours for 0.9% of the participants, 5–10 hours for 0.6% of the participants, and 10–25 hours for 0.1% of the participants. Table 1 presents characteristics of the study population. Spearman correlation coefficients of time spent in MVPA assessed by questionnaire with time spent in accelerometer-assessed MVPA short, long and ≥1 minute bouts were: 0.24 (p < 0.001), 0.24 (p < 0.001), and 0.27 (p < 0.001), respectively.

### Physical activity in successful agers and the others

Overall, successful agers practiced more MVPA than all others (unadjusted models, all p < 0.0001, Table 1). Linear multivariate regression showed successful agers to report 3.79 (95% CI: 1.39, 6.19) minutes more MVPA. Accelerometer data suggested this difference to be 3.40 (95% CI: 2.44, 4.35) minutes for MVPA undertaken in short bouts, 4.16 (95% CI: 3.11, 5.20) minutes for long bouts and 7.55 (95% CI: 5.86, 9.24) minutes for all MVPA in bouts of 1 minute or more. In addition, the intensity of MVPA as measured by mean acceleration of MVPA bouts was higher in successful agers than in the others (mean ENMO difference = 8.75 m*g*; 95% CI: 5.65, 11.85). This difference corresponds to 22% of the standard deviation of mean acceleration of MVPA bouts in the study sample.

### Proportion of successful agers by physical activity group

**Figure 1:**
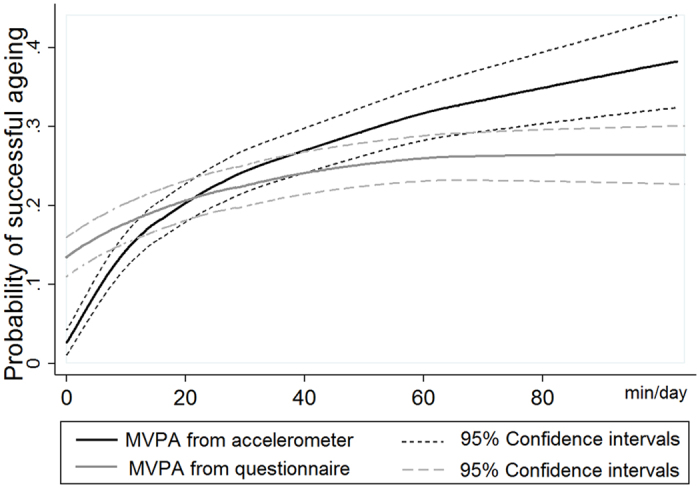
Probability of successful ageing as a function of daily duration in MVPA.

Figure 1 shows the probability of successful ageing to be higher with increasing time spent in MVPA albeit with a plateauing of effects at longer durations; associations were stronger using accelerometer- than questionnaire-assessed MVPA. This general pattern was also observed for proportion of successful agers by categories of time spent in MVPA (Table 2). Accelerometer data show that in those with daily duration of MVPA < 10 min, the prevalence of successful ageing was 9.3%; this rose to 29.5% in those with 30 min of MVPA or more per day. The corresponding estimates for questionnaire data were 14.9 and 25.8%.

Short and long bouts as defined by accelerometer data had a similar association with successful ageing: in multivariate logistic analyses, OR estimates for a 10-minute increase per day of MVPA undertaken in short and long bouts were 1.17 (95% CI: 1.09, 1.26) and 1.15 (95% CI: 1.08, 1.22), respectively (Table 2). This led us to use MVPA bouts lasting 1 minute or more as the bout metric in subsequent analysis. For every 10-minute increase in MVPA per day, the odds for successful ageing increased by 4% (95% CI: 2%, 7%) when using reported MVPA compared to 16% (95% CI: 12%, 20%) with accelerometer data – the non-overlapping confidence intervals suggest that the difference in ORs is statistically significant at p < 0.05. For accelerometer-assessed MVPA, 10–20 min and 20–30 min of MVPA per day was associated with around 2.2–2.5-fold higher odds of successful ageing compared to < 10 min MVPA. Reanalysis using 10–20 min MVPA as the reference group showed 20 min or more of MVPA to be associated with 1.38 greater odds of successful ageing (95% CI: 1.12, 1.69).

Further analysis to test whether the pattern of MVPA mattered for those undertaking the recommended ≥150 minutes of MVPA per week showed similar associations with successful ageing in those undertaking MVPA in bouts of 1 minute or more or 10 minutes or more (OR = 1.92, 95% CI: 1.60, 2.30 and 1.74, 95% CI: 1.38, 2.18 respectively). The pattern was similar, albeit associations weaker, using questionnaire-assessed MVPA (OR = 1.29, 95% CI: 1.09, 1.53, Table 2).

The odds ratio of successful ageing increased as the intensity of accelerometer assessed MVPA increased (OR = 1.16, 95% CI: 1.07, 1.26 for 1 SD (39.4 m*g*) higher acceleration). Compared to the first quartile of intensity, the 2^nd^, 3^rd^, and 4^th^ quartile of MVPA intensity were associated with an OR of successful ageing of 1.50 (95% CI: 1.17, 1.94), 1.42 (95% CI: 1.10, 1.84), and 1.75 (95% CI: 1.35, 2.27), respectively.

### Sensitivity analyses

The associations between MVPA and successful ageing were also observed when chronic disease and functioning were considered as separate outcomes (Table 3). Apart from good mental health, MVPA was linked to all functioning components, with associations being stronger for absence of disability and motor function (Table 3). For mental health, an association with MVPA was only observed for questionnaire data, possibly reflecting common method variance, and for long accelerometer-assessed bouts, possibly reflecting gains only over longer periods of MVPA. Associations with successful ageing and its components were similar when using the more stringent MVPA cut-point of 120 m*g* (Supplementary Table S1). Excluding participants who reported disability and those who died in two years following data collection from the analysis somewhat attenuated the associations in the main analysis (Supplementary Table S2).

## Discussion

This study based on 3,749 adults aged 60 to 83 years presents three key findings. First, accelerometer data show that successful agers spend on average 8 minutes more per day in MVPA than their unhealthy counterparts; both longer daily duration and higher intensity of MVPA bouts were associated with greater probability of successful ageing. Second, associations between MVPA and successful ageing were stronger with physical activity assessed by accelerometer. Third, length of a bout of MVPA, short (1–9.59 min) or long (≥10 min), did not modify the association of MVPA with successful ageing.

Our data show the prevalence of successful ageing to be higher with longer duration of MVPA, with a weakening of the effect at daily durations longer than 20 or 30 minutes. Similar findings, albeit with questionnaire-assessed physical activity and a range of health outcomes, have been reported^40^,^41^,^42^. A recent meta-analysis concluded that although more total physical activity was associated with lower risk for breast and colon cancer, diabetes, ischemic heart disease, and ischemic stroke, major gains were observed at lower levels of activity, with diminishing returns at higher levels of activity^42^. For example, compared to less active participants (<600 MET.minutes/week), the risk of colon cancer was reduced by 10%, 17% and 21% respectively in those reporting 600–3999, 4000–7999, and ≥8000 MET.minutes/week. In our study, successful ageing was twice as likely for daily MVPA of 10 to 20 minutes, compared to less than 10 minutes. However, compared to 10 to 20 minutes per day of MVPA, successful ageing was only 38% more likely in those undertaking 20 to 30 minutes of MVPA per day. Our findings on MVPA intensity are concordant; with fewer benefices at higher intensity levels. This is in agreement with recent findings suggesting that even a little MVPA is good for successful ageing^43^.

Previous studies, based on cross-sectional or longitudinal designs, have found that longer self-reported duration in MVPA was associated with greater likelihood of successful ageing^7^,^8^,^10^,^11^,^12^,^13^,^44^. Our findings add to this evidence using data on both subjective and objective assessments of MVPA. We show that the association with successful ageing was more pronounced for MVPA assessed by accelerometer compared to questionnaire. Previous studies have also shown objective assessments of physical activity to be more strongly associated with metabolic syndrome^45^, biomarkers^46^ and obesity indicators^37^. Taken together these findings suggest that measurement error inherent to self-reported physical activity measures leads to misclassification, attenuating associations with health outcomes^47^. In relation to successful ageing, it is possible that the compendium of physical activities, developed for adults up to 65 years, does not adequately reflect physical activity patterns of older adults.

Public health messages encourage people to undertake bouts of physical activity lasting a minimum of 10 minutes. However, bouts of less than 10 minutes are likely to be more achievable for older adults. We found associations with successful ageing to be similar for MVPA bouts lasting less than 10 minutes and 10 minutes or more. Few previous studies reported similar association with time spent in short and long bouts of MVPA with cardio-metabolic outcomes^20^,^21^,^22^ and multimorbidity^23^. Indeed, bouts of MVPA of at least 10 minutes are recommended for cardiorespiratory fitness^6^,^48^, although appropriate experimental studies to assess associations between duration spent in different lengths of MVPA bouts and health outcomes are lacking^48^.

Strengths of this study include its large study sample, the use of both questionnaire- and accelerometer-assessed physical activity, and high compliance for accelerometer wear among participants. Cross-sectional design is a limitation, precluding inferences about temporality, such as whether physical activity preceded successful ageing or was preceded by successful ageing. However, sensitivity analyses excluding participants with disability and those who died in 2 years after data collection suggest that our findings cannot be entirely explained by reduced physical activity in already sick participants. Furthermore, we have previously used longitudinal data, using questionnaire-assessed MVPA, to show an association of physical activity with successful ageing^13^. Future longitudinal studies using accelerometers are needed to confirm the findings in the present study. Second, although the sample covered a wide socioeconomic range, data are from an occupational cohort and are not representative of the general population (i.e. composed of more men and highly educated participants). In addition, the analytic sample was composed of the more physically active participants, who were more likely to age successfully. Taken together, these aspects would lead to be an underestimation of the association between MVPA and successful ageing.

In conclusion, accelerometer data from a large sample of UK older adults suggest that successful agers practiced MVPA for longer duration and at higher intensity. Higher proportion of successful agers was also observed in those practicing more MVPA, independently of the length of the MVPA bout. Future studies using a longitudinal design should examine whether physical activity predicts successful ageing over time.

## Additional Information

**How to cite this article:** Menai, M. *et al*. Accelerometer assessed moderate-to-vigorous physical activity and successful ageing: results from the Whitehall II study. *Sci. Rep.* **7**, 45772; doi: 10.1038/srep45772 (2017).

**Publisher's note:** Springer Nature remains neutral with regard to jurisdictional claims in published maps and institutional affiliations.

## Supplementary Material

Supplementary Material

## Figures and Tables

**Table 1:** Characteristics of the study population.

	Successful agers	Unsuccessful agers	P (chi-2 or t-test)
	N (%)	N (%)	
N	789 (21.1)	2,960 (79.9)	
Age (years), M(SD)	68.2 (5.4)	69.7 (5.7)	<0.0001
Women	212 (26.9)	741 (25.0)	0.29
Ethnicity (White)	786 (99.6)	2,690 (90.9)	<0.0001
High Occupational position at age 50y	464 (58.8)	1,196 (40.4)	<0.0001
University or higher	314 (39.8)	827 (27.9)	<0.0001
Smoking			
Current	21 (2.7)	186 (6.3)	<0.0001
Ex	343 (43.5)	1,374 (46.4)	
Never	425 (53.9)	1,400 (47.3)	
Alcohol			
None	110 (13.9)	633 (21.4)	<0.0001
Moderate	552 (70.0)	1,925 (65.0)	
High	127 (16.1)	402 (13.6)	
Fruit and vegetable consumption			
Less than daily	116 (14.7)	654 (22.1)	<0.0001
Daily	139 (17.6)	664 (22.4)	
At least twice daily	534 (67.7)	1,642 (55.5)	
Minutes per day of MVPA, M(SD)			
Assessed by questionnaire	35.5 (34.0)	29.2 (30.3)	<0.0001
Assessed by accelerometer			
MVPA 1 + min bouts	34.9 (25.7)	24.5 (21.6)	<0.0001
MVPA 10 + min bouts	13.3 (18.3)	7.6 (11.9)	<0.0001
150 minutes per week of MVPA			
Assessed by questionnaire	479 (60.7)	1,487 (50.5)	<0.0001
Assessed by accelerometer			
MVPA 1 + min bouts	518 (65.7)	1,345 (45.4)	<0.0001
MVPA 10 + min bouts	149 (18.9)	283 (9.6)	<0.0001
Acceleration of MVPA 1 + min bouts, M(SD)	186.3 (46.8)	175.8 (36.9)	<0.0001
Chronic diseases			
CHD	0	572 (19.3)	
Stroke	0	40 (1.4)	
Diabetes	0	469 (16.3)	
Cancer	0	528 (17.9)	
Neurodegenerative diseases	0	24 (0.8)	
Functioning			
No disability	789 (100)	2,551 (87.4)	
Standardized cognitive score, M(SD)	0.6 (0.6)	−0.1 (1.0)	
Walking speed (m/s), M(SD)	1.3 (0.2)	1.1 (0.3)	
FEV1/height^2^ (L/m^2^), M(SD)	1.1 (0.2)	0.9 (0.2)	
Good mental health (CESD < 16)	789 (100)	2,378 (85.7)	

**Table 2:** Association between daily duration of MVPA and weekly recommendation with successful ageing.

	QUESTIONNAIRE ASSESSED MVPA					ACCELEROMETER ASSESSED MVPA				
	Median duration (min/day)	N	% Successful agers	OR	95% CI	Median duration (min/day)	N	% Successful agers	OR	95% CI
**Daily duration of MVPA per 10 min increase**	23.0	3,749	21.05	**1.04**	**1.02, 1.07**					
Short bouts		—	—	—		15.7	3,749	21.05	**1.17**	**1.09, 1.26**
Long bouts		—	—	—		3.7	3,749	21.05	**1.15**	**1.08, 1.22**
≥1 min bouts		—	—	—		21.3	3,749	21.05	**1.16**	**1.12, 1.20**
**Daily duration of MVPA**										
<10 min	2.7	1,192	14.93	1	(Ref.)	4.8	912	9.32	1	(Ref.)
10 to 20 min	15.0	558	22.04	**1.46**	**1.12, 1.91**	14.8	865	19.42	**2.18**	**1.63, 2.91**
20 to 30 min	25.7	473	19.87	1.21	0.91, 1.62	24.5	682	22.87	**2.47**	**1.83, 3.35**
≥30 min per day	50.9	1,526	25.82	**1.63**	**1.32, 2.02**	45.0	1,290	29.46	**3.32**	**2.51, 4.38**
**Recommendation of 150 min per week**										
No	5.4	1,766	17.55	1	(Ref.)	10.4	1,886	14.37	1	(Ref.)
Yes	42.9	1,966	24.36	**1.29**	**1.09, 1.53**	36.6	1,863	27.80	**1.92**	**1.60, 2.30**

**Table 3:** Association of daily duration and weekly recommendation of MVPA with components of successful ageing.

		Better functioning					
	No chronic disease	Overall	No disability	Better cognitive function	Better motor function	Better respiratory function	Good mental health
	OR (95% CI)	OR (95% CI)	OR (95% CI)	OR (95% CI)	OR (95% CI)	OR (95% CI)	OR (95% CI)
**QUESTIONNAIRE ASSESSED PHYSICAL ACTIVITY**							
** Daily duration of MVPA per 10 min increase**	**1.03 (1.01–1.06)**	**1.05 (1.02–1.07)**	**1.12 (1.07–1.17)**	1.01 (0.98–1.03)	**1.08 (1.05–1.11)**	**1.04 (1.01–1.07)**	**1.13 (1.07–1.18)**
**Daily duration of MVPA**							
<10 min	1 (Ref.)	1 (Ref.)	1 (Ref.)	1 (Ref.)	1 (Ref.)	1 (Ref.)	1 (Ref.)
10 to 20 min	1.23 (0.98–1.53)	**1.54 (1.21–1.97)**	**1.67 (1.19–2.33)**	1.20 (0.94–1.53)	**1.44 (1.15–1.80)**	1.12 (0.89–1.42)	**1.49 (1.08–2.05)**
20 to 30 min	1.04 (0.83–1.31)	**1.34 (1.04–1.73)**	**1.61 (1.13–2.29**	1.14 (0.88–1.47)	**1.38 (1.09–1.75)**	1.23 (0.95–1.58)	**1.49 (1.06–2.10)**
≥30 min per day	**1.35 (1.13–1.60)**	**1.72 (1.42–2.08)**	**2.58 (1.95–3.41)**	1.19 (0.98–1.44)	**1.75 (1.46–2.09)**	**1.39 (1.16–1.68)**	**2.24 (1.70–2.94)**
**Recommendation of 150 min per week**							
No	1 (Ref.)	1 (Ref.)	1 (Ref.)	1 (Ref.)	1 (Ref.)	1 (Ref.)	1 (Ref.)
Yes	**1.15 (1.00–1.34)**	**1.35 (1.15–1.58)**	**1.92 (1.52–2.42)**	1.10 (0.94–1.29)	**1.43 (1.23–1.66)**	**1.29 (1.11–1.51)**	**1.72 (1.37–2.15)**
**ACCELEROMETER ASSESSED PHYSICAL ACTIVITY**							
**Daily duration of MVPA per 10 min increase**							
Short bouts	**1.24 (1.16–1.34)**	**1.16 (1.08–1.24)**	**1.53 (1.34–1.76)**	**1.10 (1.03–1.19)**	**1.28 (1.19–1.38)**	**1.16 (1.08–1.24)**	0.97 (0.88–1.08)
Long bouts	1.06 (0.98–1.13)	**1.13 (1.06–1.20)**	1.15 (0.99–1.34)	1.01 (0.95–1.08)	**1.12 (1.04–1.21)**	1.06 (0.99–1.13)	**1.12 (1.00–1.26)**
≥1 min bouts	**1.15 (1.10–1.19)**	**1.14 (1.10–1.18)**	**1.35 (1.25–1.47)**	**1.06 (1.02–1.10)**	**1.20 (1.15–1.25)**	**1.10 (1.06–1.15)**	1.04 (0.99–1.10)
**Daily duration of MVPA bouts ≥1 min**							
<10 min	1 (Ref.)	1 (Ref.)	1 (Ref.)	1 (Ref.)	1 (Ref.)	1 (Ref.)	1 (Ref.)
10 to 20 min	**1.52 (1.25–1.86)**	**1.87 (1.46–2.39)**	**2.24 (1.68–2.99)**	**1.52 (1.21–1.90)**	**1.96 (1.59–2.41)**	**1.26 (1.01–1.57)**	1.35 (0.99–1.85)
20 to 30 min	**1.63 (1.31–2.03)**	**2.51 (1.94–3.25)**	**4.21 (2.84–6.22)**	**1.53 (1.20–1.96)**	**2.06 (1.64–2.59)**	**1.72 (1.35–2.19)**	1.38 (0.98–1.94)
≥30 min per day	**2.30 (1.88–2.82)**	**2.71 (2.14–3.44)**	**3.87 (2.80–5.34)**	**1.67 (1.34–2.08)**	**3.08 (2.49–3.80)**	**1.68 (1.36–2.09)**	1.32 (0.98–1.79)
**Recommendation of 150 min per week**							
**MVPA bouts ≥1 min**							
No	1 (Ref.)	1 (Ref.)	1 (Ref.)	1 (Ref.)	1 (Ref.)	1 (Ref.)	1 (Ref.)
Yes	**1.64 (1.41–1.90)**	**1.81 (1.54–2.13)**	**2.41 (1.87–3.10)**	**1.31 (1.11–1.54)**	**1.82 (1.56–2.13)**	**1.51 (1.29–1.77)**	1.12 (0.89–1.41)
**MVPA bouts ≥10 min**							
No	1 (Ref.)	1 (Ref.)	1 (Ref.)	1 (Ref.)	1 (Ref.)	1 (Ref.)	1 (Ref.)
Yes	**1.57 (1.23–2.01)**	**1.54 (1.24–1.92)**	**2.09 (1.29–3.38)**	1.01 (0.79–1.29)	**1.94 (1.50–2.52)**	**1.34 (1.05–1.71)**	1.09 (0.75–1.57)
