# Supplementary material for: Accelerometer assessed moderate-to-vigorous physical activity and successful ageing: results from the Whitehall II study
Source: Sci Rep. 2017 Apr 3;7:45772. doi: 10.1038/srep45772 (PMC5377945; doi:10.1038/srep45772)
Supplement: Supplementary Material [file srep45772-s1.pdf]

Supplementary Information

Accelerometer assessed moderate-to-vigorous physical activity and successful ageing: results from the Whitehall II study

Mehdi Menai PhD[a], Vincent T. van Hees PhD[b], Alexis Elbaz MD, PhD[a], Mika Kivimaki PhD[c], Archana Singh-Manoux PhD[a,c], Séverine Sabia PhD[a, c*]

Supplementary Methods. Definition of MVPA bouts

Physical activity is considered as moderate-to-vigorous when the associated metabolic equivalent (MET) equals to 3 or above. There is currently no recommendation available for MVPA cut-point using wrist acceleration in elderly population. So we used data from a laboratory experiment based on 30 adults who walked at 3 km/h and 5 km/h[29]. As walking at 4km/h is considered a moderate physical activity[27], we choose a cut-point equal to the mean wrist-acceleration plus its standard deviation for walking at 3 km/h (100 m*g*). This cut-point has been used in previous publications using the Whitehall II cohort[36,37] and other studies[28-30].

An alternative cut-point of 120 m*g* was used in sensitivity analyses as this was the mid-point between mean acceleration for walking at 3 km/h and 5 km/h. This 120 m*g* cut-point is more stringent, and captures activity which is more likely to be within the more intense and not in the less intense moderate-to-vigorous range, but reducing the possibility of contamination by light activity.

In order to account for time spent in short MVPA bouts (1 to 9.59 minutes), consecutive 5s-epoch mean acceleration (expressed as the Euclidean norm (magnitude) of the 3 raw signals minus 1 *g* (ENMO)) needed to be at 100 m*g* or above for at least 1 minute and less than 10 minutes with allowance of no more than 20% (no more than two 5s-epochs) of a 1-min moving window (12 consecutive 5s-epochs) below the threshold. Time spent in long MVPA bouts (≥10 minutes) accounted for activity periods for which consecutive 5s-epoch mean acceleration were at 100m*g* or above for at least 10 minutes, with allowance of 20% (twenty-four 5s-epochs) of a 10-min moving window (120 consecutive 5s-epochs) below the threshold, each interruption lasting one minute (12 consecutive 5s-epcoh) at most. Duration of each MVPA bout was calculated as the time elapsed between the start (beginning of first epoch above the threshold) and the end (end of the last epoch above the threshold) of the bout.

Supplementary Figure S1. Flow Chart of the Study

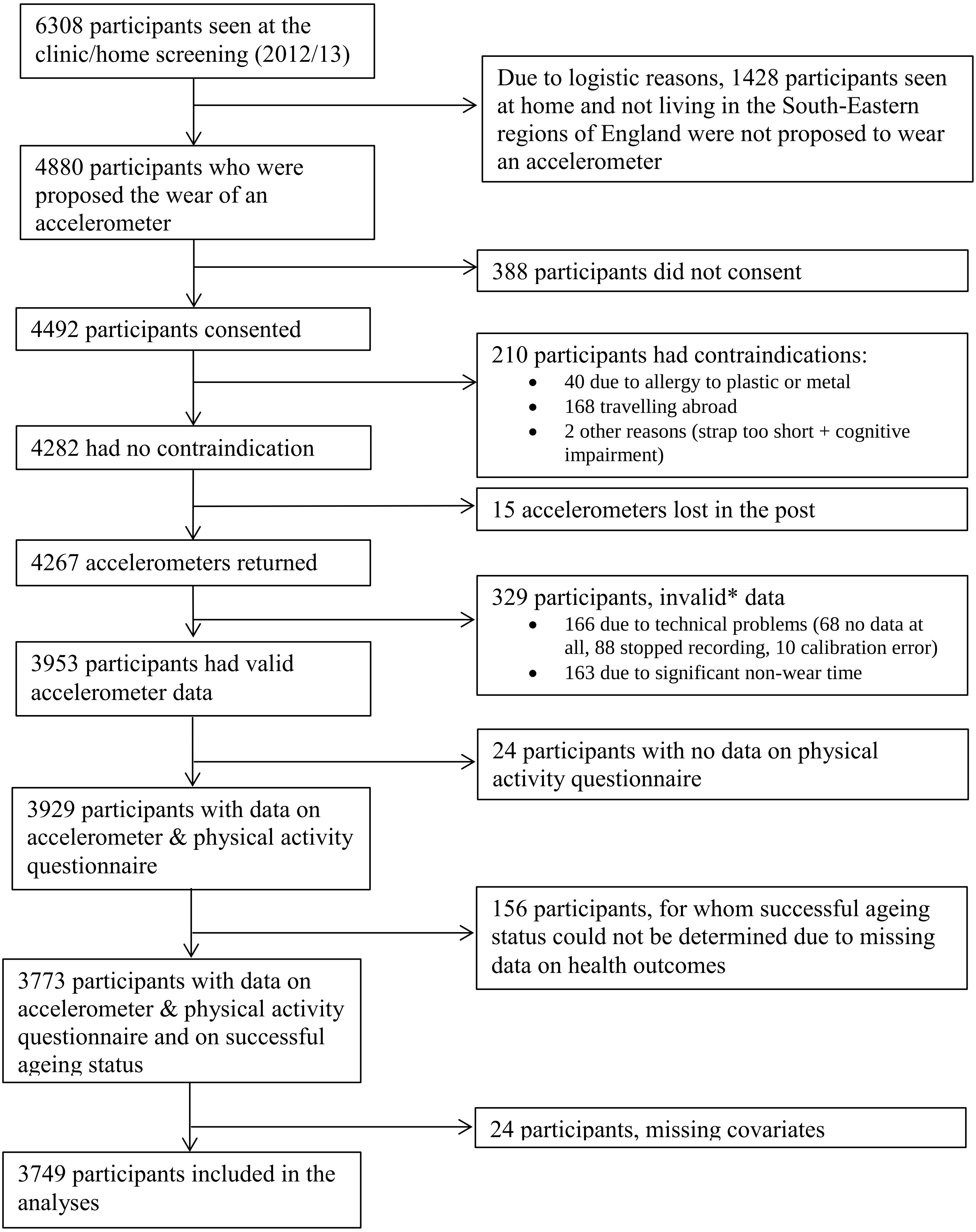

Supplementary Table S1. Association between daily duration of MVPA and weekly recommendation with successful ageing excluding participants reporting disability and those who died in the couple of years following the examination (N=3,323)

	**QUESTIONNAIRE ASSESSED MVPA**					**ACCELEROMETER ASSESSED MVPA**				
	**Median duration (min/day)**	**N**	**% Successful agers**	**OR**	**95% CI**	**Median duration (min/day)**	**N**	**% Successful agers**	**OR**	**95% CI**
**Daily duration of MVPA per 10 min increase**	25.2	3,323	21.38	**1.04**	**1.01, 1.06**					
Short bouts		-	-	**-**		16.5	3,323	21.38	**1.15**	**1.06, 1.24**
Long bouts		-	-	**-**		4.1	3,323	21.38	**1.14**	**1.07, 1.22**
≥ 1 min bouts		-	-	**-**		22.4	3,323	21.38	**1.14**	**1.10, 1.19**
**Daily duration of MVPA**										
< 10 min	2.7	993	17.93	1	(Ref.)	5.3	707	12.02	1	(Ref.)
10 to 20 min	15.0	497	24.55	**1.37**	**1.04, 1.80**	14.8	767	21.64	**1.88**	**1.40, 2.53**
20 to 30 min	25.7	422	22.27	1.11	0.83, 1.50	24.5	642	24.30	**2.07**	**1.52, 2.81**
≥30 min per day	50.9	1,411	27.78	**1.47**	**1.18, 1.82**	45.4	1,207	31.40	**2.80**	**2.11, 3.72**
**Recommendation of 150 min per week**										
No	5.4	1,503	20.56	1	(Ref.)	11.1	1,579	17.04	1	(Ref.)
Yes	42.9	1,804	26.44	**1.19**	**1.00, 1.42**	36.6	1,744	29.64	**1.78**	**1.48, 2.14**

Supplementary Table S2. Associations between daily durations of MVPA and recommendation with successful ageing, absence of chronic disease and good functional status for 120 mg threshold to define MVPA.

	Successful ageing	Successful ageing: components	
		No chronic disease*	Better functioning†
	OR‡ (95% CI)	OR‡ (95% CI)	OR‡ (95% CI)
**Daily duration of MVPA per 10 min increase**			
Short bouts	**1.25 (1.14, 1.38)**	**1.32 (1.20, 1.46)**	**1.22 (1.12, 1.34)**
Long bouts	1.17 (1.08, 1.28)	1.06 (0.96, 1.16)	**1.15 (1.06, 1.25)**
**Daily duration of MVPA per 10 min increase**			
≥ 1 min bouts	**1.21 (1.15, 1.27)**	**1.19 (1.13, 1.25)**	**1.18 (1.13, 1.24)**
**Daily duration of MVPA bouts ≥ 1 min**			
< 10 min	Referent	Referent	Referent
10 to 20 min	**1.88 (1.50, 2.36)**	**1.37 (1.15, 1.65)**	**1.86 (1.52, 2.27)**
20 to 30 min	**2.18 (1.69, 2.81)**	**1.48 (1.19, 1.84)**	**2.15 (1.70, 2.71)**
≥ 30 min per day	**2.66 (2.09, 3.40)**	**2.10 (1.68, 2.63)**	**2.29 (1.83, 2.86)**
**Recommendation of 150 min per week**			
**MVPA bouts ≥ 1 min**			
No	Referent	Referent	Referent
Yes	**1.64 (1.38, 1.96)**	**1.56 (1.32, 1.85)**	**1.49 (1.26, 1.75)**
**MVPA bouts ≥ 10 min**			
No	Referent	Referent	Referent
Yes	**2.11 (1.58, 2.83)**	**1.98 (1.39, 2.83)**	**1.76 (1.32, 2.35)**

* Defined as absence of coronary artery disease, stroke, diabetes, depression, cancer and dementia.

† Defined as absence of any disabilities, being above the sex and age specific 1st tertile of cognitive, walking and respiratory score of the population and having a CESD score <16.

‡ Logistic regressions adjusted for age, gender, ethnicity, education, smoking status, alcohol consumption, fruit and vegetable consumption, season and accelerometer wear time.

MVPA: Moderate to vigorous physical activity. Ref: reference.

N cases/ N= 789/3,762 for successful ageing, 2,361/3,714 for absence of chronic disease and 1,082/3,658 for good functional status.
